# ^1^H NMR Metabolic Fingerprinting to Probe Temporal Postharvest Changes on Qualitative Attributes and Phytochemical Profile of Sweet Cherry Fruit

**DOI:** 10.3389/fpls.2015.00959

**Published:** 2015-11-10

**Authors:** Vlasios Goulas, Ioannis S. Minas, Panayiotis M. Kourdoulas, Athina Lazaridou, Athanassios N. Molassiotis, Ioannis P. Gerothanassis, George A. Manganaris

**Affiliations:** ^1^Department of Agricultural Sciences, Biotechnology and Food Science, Cyprus University of TechnologyLemesos, Cyprus; ^2^Nuclear Magnetic Resonance Center, Department of Chemistry, University of IoanninaIoannina, Greece; ^3^School of Agriculture, Aristotle University of ThessalonikiThessaloniki, Greece

**Keywords:** postharvest physiology, mechanical properties, Nuclear Magnetic Resonance, metabolic profile, antioxidants, *Prunus avium*

## Abstract

Sweet cherry fruits (*Prunus avium* cvs. ‘Canada Giant’, ‘Ferrovia’) were harvested at commercial maturity stage and analyzed at harvest and after maintenance at room temperature (storage at ∼20°C, shelf life) for 1, 2, 4, 6, and 8 days, respectively. Fruit were initially analyzed for respiration rate, qualitative attributes and textural properties: ‘Canada Giant’ fruit were characterized by higher weight losses and stem browning index, being more intense over the late stages of shelf life period; meanwhile ‘Ferrovia’ possessed appreciably better performance even after extended shelf life period. A gradual decrease of respiration rate was monitored in both cultivars, culminated after 8 days at 20°C. The sweet cherry fruit nutraceutical profile was monitored using an array of instrumental techniques (spectrophotometric assays, HPLC, ^1^H-NMR). Fruit antioxidant capacity was enhanced with the progress of shelf life period, concomitant with the increased levels of total anthocyanin and of phenolic compounds. ‘Ferrovia’ fruit presented higher contents of neochlorogenic acid and *p*-coumaroylquinic acid throughout the shelf life period. We further developed an ^1^H-NMR method that allows the study of primary and secondary metabolites in a single running, without previous separation and isolation procedures. Diagnostic peaks were located in the aliphatic region for sugars and organic acids, in the aromatic region for phenolic compounds and at 8.2–8.6 ppm for anthocyanins. This NMR-based methodology provides a unifying tool for quantitative and qualitative characterization of metabolite changes of sweet cherry fruits; it is also expected to be further exploited for monitoring temporal changes in other fleshy fruits.

## Introduction

Sweet cherry (*Prunus avium* L.) is a highly appreciated fresh product; its consumer acceptance is largely based on overall appearance defined as skin and stem color, and the absence of other physical defects and on taste, defined as the ratio of total soluble solids concentration (SSC) to acidity (Crisosto et al., 2003; Serrano et al., 2005a). However, sweet cherry is a highly perishable commodity, which in some cases, does not reach in the consumer at the optimal quality after transport and/or marketing (Remon et al., 2003; Serrano et al., 2005a,b). Fruit is characterized by high respiration and water loss rates that favor rapid postharvest deterioration with detrimental effects on appearance (Belge et al., 2014). In addition, sweet cherry fruits are particularly vulnerable to physiological disorders, as well as to fungal rots, all of which limit their shelf life (Alique et al., 2005).

Apart from its qualitative attributes, sweet cherry is also considered a reservoir of bioactive compounds, primarily anthocyanins, and hydroxycinnamic acids. Phenolic compounds, apart from their health-promoting properties, play a key role on sweet cherry quality attributes since they contribute to color and flavor. Despite its significant economic impact as a temperate fruit crop, few comprehensive studies dealt with physicochemical and phytochemical aspects of different cultivars (Ballistreri et al., 2013). Most of these studies have focused on analysis solely at harvest time, although alterations in the phytochemical content during the postharvest period have been also reported (Valero et al., 2011).

In the current study, a comprehensive appraisal of postharvest sweet cherry fruit quality and its phytochemical profile on two cultivars with distinct profiles was performed. Temporal changes of physiological, physicochemical, and antioxidant properties were further monitored by Nuclear Magnetic Resonance (NMR) spectroscopy that allowed a rapid screening of specific primary and secondary metabolites of sweet cherries.

## Materials and Methods

### Fruit Material and Sampling Process

‘Canada Giant’ and ‘Ferrovia’ sweet cherry fruits were harvested at commercial maturity stage from a commercial orchard (Agios Georgios, Central Macedonia, Greece) based on size and background color. Fruit were immediately transferred to the Laboratory of Pomology (Aristotle University of Thessaloniki), separated to distinct categories based on fruit size and the most representative lots were used for further studies; simultaneously, sorting for major defects such as decay or cracking was carried out. Such cultivars were selected based on their distinct anatomical properties: ‘Canada Giant’ fruit is characterized by shorter stem and bigger dimensions compared to ‘Ferrovia’ (**Supplementary Figure S1**). Fruit were randomly divided into six treatment units per cultivar; each treatment unit included 60 sweet cherries and analyzed at harvest (0 day) and after maintenance at ∼20°C for 1, 2, 4, 6, and 8 days, to simulate shelf life conditions and probe the evolution of postharvest changes.

Thirty fruits, divided into three 10-fruit sub-lots, were initially analyzed for textural properties. Subsequently, the same fruits were used for SSC and titratable acidity (TA) determination. Another lot of 30 fruits, divided into three 10-fruit sub-lots, and after removal of pit were immediately frozen in liquid nitrogen and stored at –20°C, until needed. Another two lots, of 30 fruits each, were used non-destructively during the 8-days shelf life period. One lot was used for determination of external color, weight loss, and subjective determination of stem color, fruit cracking, shriveling and pitting, and overall acceptance; the other lot was used for respiration rate determination.

### Qualitative Properties

Thirty fruits were evaluated non-destructively for weight loss, color parameters and subjective qualitative analysis. The weight loss was calculated by the equation: weight loss (%) = (W_o_ - W_f_/W_o_) × 100, where W_o_ is the initial weight and W_f_ is the final weight.

External color was measured objectively with a Minolta CR200 colorimeter (Minolta, Osaka, Japan) and the CIE (Commission Internationale de l’Eclairage) parameters (*L^∗^, a^∗^, b^∗^*), *C^∗^* (chroma), and *h^o^* (hue angle) were determined in 30 fruits per treatment, using two surfaces on the opposite sides of each fruit at the equator. Values of *L^∗^, a^∗^*, and *b^∗^* were measured to describe a three-dimensional color space in which *L^∗^* indicates lightness read from 0 (completely opaque or “black”) to 100 (completely transparent or “white”). A positive *a^∗^* value indicates redness (–*a^∗^* is greenness) and a positive *b^∗^* value yellowness (–*b^∗^* is blueness) on the hue-circle. The hue angle (°), hue = arctg (*b^∗^*/*a^∗^*), expresses the color nuance ranging from red-purple: (0°), yellow (90°), bluish-green (180°), and blue (270°). The chroma, obtained as (*a^∗^*^2^ + *b^∗^*^2^)^1/2^, is measure of chromaticity (*C^∗^*), which denotes the purity or saturation of the color (Gonçalves et al., 2007).

Soluble solids concentration was determined in the juice from 10 fruit from each sub-lot with a digital refractometer (Atago PR-101, Atago Co. Ltd., Japan). TA was determined by potentiometric titration with 0.1 N NaOH up to pH 8.2, using 5 mL of diluted juice in 20 mL distilled H_2_O and results expressed as % malic acid. The ripening index (RI) was calculated by the ratio of SSC to TA.

Subjective determination of quality attributes (stem color, surface pitting, and overall acceptance) was conducted according to Feng et al. (2004) as follows: Stem color: 0:Green, fresh appearance, less than 30% brown, 1: Substantially green with 30–50% brown, 2: Substantially brown with less than 50% green, Surface pitting: 0: None or less than 0.3 cm^2^ pitting, 1: Pitting affecting 0.3–0.5 cm^2^, 2: Pitting affecting greater than 0.5 cm^2^, Overall acceptability: 0: Good commercial quality, 1: Some damage, but still commercially salable, 2: Not commercially salable.

For those subjective quality factors, an index was used to express a single quality grade for each quality attribute for each replicate using the following formula: Index = (number of cherries given score 2 × 1.0) + (number of cherries given score 1 × 0.5)/total number of cherries evaluated.

### Respiration Rate

Five batches of 10 fruit from each sub-lot were enclosed in a 0.5 L airtight jars for 30 min at 20°C. Respiration was assessed by CO_2_ production in the gas phase of the jars, measured automatically, using a 280 Combo infrared gas analyser (David Bishop Instruments, Heathfield, UK) connected to a computer and expressed as CO_2_ mL kg^-1^ h^-1^.

### Textural Properties

For each cultivar, the textural properties of thirty fruits were determined using a TA-XT2i Texture Analyzer (Stable Microsystems, Godalming, Surrey, UK) interfaced to a personal computer. Fruit firmness was measured using a flat steel plate (75 mm diameter) mounted on the machine; the fruit was placed with its large dimension on the ‘crisp fracture support ring’ (Stable Microsystems). Then the force that is required for a 15% deformation of the fruit height was recorded; the speed of the compression plunger was 0.8 mm s^-1^. Normalized firmness force was calculated as the ratio of the force required to achieve 15% fruit deformation over the fruit large diameter (Newtons mm^-1^). For the stem traction force, a tensile grip was used to remove the stem and results were expressed in Newtons; the speed of the tensile test was also at 0.8 mm s^-1^. The fruit was placed beneath a plate having a round hole (10 mm diameter) through which the stem was directed to the grip. The skin penetration force was finally measured using a 2 mm diameter stainless steel needle mounted on the machine and inserted into the cherry flesh at a 0.8 mm s^-1^ speed, and the maximum force recorded during the penetration (5 mm depth) was expressed in Newtons. The fruit was mounted on the ‘crisp fracture support ring’.

### Extraction of Phenolic Compounds

The frozen fruit material (5 g) was homogenized in a Polytron (2 min on ice) with 10 mL of extraction solution (water/methanol 2:8 containing 2 mM NaF to inactivate polyphenol oxidases and prevent phenolic degradation due to browning). Homogenates were kept in ice until centrifuged (4700 rpm, 15 min, 4°C); the supernatant was recovered carefully to prevent contamination with the pellet. An aliquot of extract was filtered through a 0.45-μm filter and directly analyzed by HPLC (Waters 1525, Waters Corporation, Milford, Ireland) for hydroxycinnamates and flavonols. The rest of extract was used for spectrophotometric assays or lyophilized to obtain a dry extract for NMR analysis.

### Determination of Total Phenolics and Total Anthocyanins

The amount of total phenolics was determined according to the Folin–Ciocalteu method (Goulas and Manganaris, 2011). The reaction mixture consisted of 0.5 mL of diluted extract, 5 mL of distilled water and 0.5 mL of the Folin–Ciocalteu reagent. After a period of 3 min, 1 mL of saturated sodium carbonate solution was added. Finally, the mixture was diluted to final volume of 10 ml and allowed to stand for 1 h at room temperature. The absorbance was measured at 725 nm (Lambda 25, Perkin Elmer). Each measurement was repeated in triplicate and the total phenolic content was expressed as equivalents of mg gallic acid per 100 g fresh fruit weight (FW).

The total anthocyanin content was estimated by the pH-differential assay (AOAC method 2005.02) using two buffer systems: potassium chloride buffer (0.025 M) at pH 1.0 and sodium acetate buffer (0.4 M) at pH 4.5. Samples were diluted in pH 1.0 and pH 4.5 buffers and then their absorbance were measured at 520 and 700 nm. The anthocyanin pigment concentration was calculated as cyanidin-3-*O*-glucoside equivalents. All measurements were done in triplicate and the means were reported.

### Determination of *In Vitro* Antioxidant Capacity

Determination of 2,2-diphenyl-1-picrylhydrazyl radical (DPPH) scavenging activity was performed according to Goulas and Manganaris (2011). Two milliliter of diluted extract were mixed with 1 mL of 0.3 mmol L^-1^ solution of DPPH in methanol, incubated in the dark for 30 min and the absorbance of the mixture was monitored at 517 nm. Determination of total antioxidant capacity by the ferric reducing/antioxidant power (FRAP) assay was performed according to Goulas and Manganaris (2011) as follows: a sample containing 3 mL of freshly prepared FRAP solution (0.3 mol L^-1^ acetate buffer (pH 3.6) containing 10 mmol L^-1^ 2,4,6-tripyridyl-s-triazine (TPTZ) and 40 mmol L^-1^ FeCl_3_ 10H_2_O) and 100 μL of extract was incubated at 37°C for 4 min and the absorbance was measured at 593 nm. The determination of 2,2′-azino-bis-(3-ethylbenzothiazoline-6-sulfonic acid; ABTS^∗+^) scavenging activity was carried out according to Shanmugam et al. (2007). The ABTS radical was formed from the reaction of 2.45 mM potassium persulfate with 7 mM ABTS stock solution, kept in the dark and at room temperature for 16 h. Then, ABTS radical was diluted in ethanol until a solution with absorbance of 0.700 nm ± 0.005 at 734 nm was obtained. A 200 μL aliquot of diluted extract was then homogenized with 1.8 mL of the ABTS radical. Absorbance of the samples was read at 734 nm after 6 min of reaction. The results for all three assays were expressed as μmol Trolox 100 g^-1^ FW.

### Sample Preparation for NMR Analysis

Approximately 80 mg freeze-dried extract was transferred into a 2 mL Eppendorf tube, to which 600 μL D_2_O containing 0.005% (w/v) TSPA-*d_4_*, were added. Then, the pH was adjusted at 3.0, using 1 M deuterium chloride or 1 M sodium deuteroxide. The mixture was subsequently transferred to a 5 mm NMR tube and used for the NMR analyses. The NMR experiments were performed on a Bruker AV-500 spectrometer equipped with a TXI cryoprobe; the NMR system was controlled by the software TopSpin 2.1. Chemical shifts were reported with respect to the resonance of the solvent TSPA-*d_4_*- at 0.0 ppm. ^1^H-NMR spectra were collected with a relaxation delay of 5 s and an acquisition time of 4.3 s; 64 K data points were collected and the data were treated using a line broadening exponential function of 0.3 Hz. Two hundred fifty six repetitions were used in total.

### Statistical Analysis

Values were analyzed using the general linear model or analysis of variance and the least significant difference (LSD) mean separation procedures of SPSS (SPSS v20.0., Chicago, USA).

## Results and Discussion

### Quality Attributes

**FIGURE 1:**
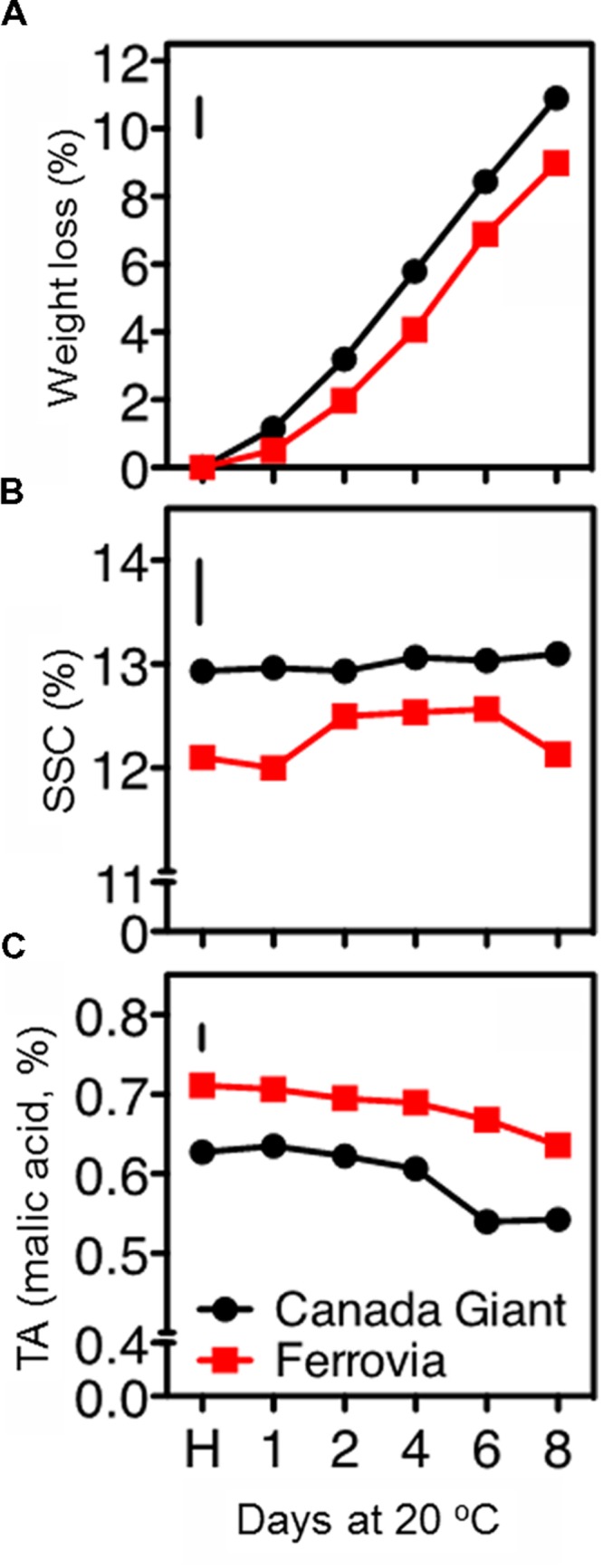
**Weight loss **(A)**, soluble solids concentration (SSC; **B**), and titratable acidity (TA), **(C)** in sweet cherry fruits (cvs. ‘Canada Giant’, ‘Ferrovia’) at harvest (H) and after additional maintenance at room temperature (20°C, shelf life) for 1, 2, 4, 6, and 8 days.** Each value represents the mean of three replications of 10 fruits analyzed at each time interval after harvest. The vertical bars in each particular figure represent the least significant difference (LSD, *P* = 0.05).

Sweet cherries are highly perishable, containing about 90% water, which is lost rapidly; dehydration is additionally leading to fruit softening and stem darkening (Drake et al., 1988). In this study, ‘Canada Giant’ fruits displayed higher weight losses compared to those of ‘Ferrovia’; a 10.9% weight loss was monitored after 8 days shelf life (**Figure 1A**), leading to both quantitative and qualitative losses.

The SSC remained invariable for both cultivars throughout the shelf life period, ranging between 12.9–13.9% and 12.0–12.6% for ‘Canada Giant’ and ‘Ferrovia’ fruit, respectively (**Figure 1B**). Bernalte et al. (2003) has also reported relatively low variations in SSC of sweet cherry fruit after harvest. Instead, great differences regarding the SSC among sweet cherry cultivars have been shown in other works, ranging between 13.5–22.7% (Ballistreri et al., 2013) and 11–25% (Martinez-Romero et al., 2006). Such differences in SSC, even for the same cultivar considered, can be attributed to microclimatic conditions, rootstock selection and planting system, as well as differences in the physiological stage adopted as a harvesting criterion.

A slight decrease of TA with the progress of shelf life period was monitored in both cultivars. ‘Ferrovia’ fruit possessed TA values among 0.64–0.71% (**Figure 1C**); Ballistreri et al. (2013) has reported higher SSC and TA contents for ‘Ferrovia’ fruits and a Ripening index (RI = SSC/TA ratio) of 22.1 at harvest. In the current study, RI was in the range 17.0–19.1 for ‘Ferrovia’ fruit, while a higher RI was found for ‘Canada Giant’ fruits (20.4–24.2). It should be also reported that TA is a highly cultivar dependent parameter, with values in the range 0.4–1.5% (Esti et al., 2002; Bernalte et al., 2003; Ballistreri et al., 2013).

All color parameters went descending with the progress of shelf life period for both cultivars (**Supplementary Table S1**), indicative of advancing senescence stage. It is worthy to note that values reported herein for *L^∗^, a^∗^, b^∗^* parameters at harvest are substantially higher than those reported for the same cultivar in another study (Ballistreri et al., 2013); this may partially explain the differences in the SSC and TA monitored (**Figure 1C**). Many studies have reported a decline in CIELab parameters (*L^∗^, a^∗^, b^∗^, C^∗^* και *h^o^*), following cold storage or maintenance at room temperature (Drake and Elfving, 2002; Serrano et al., 2005a; Gonçalves et al., 2007). The lowest values of lightness (*L*^∗^), yellowness (*b*^∗^), chroma and hue angle corresponded to the fruit of the later stages of shelf life period, having the highest anthocyanin content, as previously reported (Gonçalves et al., 2007). It seems logic that these parameters may correlate negatively with the anthocyanins levels, but it is more complex to understand why an increase in red pigments gives lower redness value readings (*a*^∗^). This phenomenon was discussed and was presumed to occur when increased pigment concentration both darkens the sample (e.g., the fruit or the fruit beverage) and increases the chroma (Eagerman et al., 1973; Gonçalves et al., 2007). When this occurs, the color scales are no longer tied linearly to the luminous transmittance (Eagerman et al., 1973). Herein, the anthocyanins did indeed darken the cherries as their concentration increased, and therefore the chromaticity responses were no longer linear, and might, in fact, have reverted to correlate negatively (Gonçalves et al., 2007). In particular for ‘Ferrovia’ fruit, a substantial decrease in hue angle with the progress of shelf life period has been elsewhere described (Alique et al., 2005). Interestingly, all color parameters were of higher value in the ‘Canada Giant’ at harvest; yet similar values in both cultivars were found after 8 days of shelf life.

Subjective quality assessment indicated a higher stem browning index for the ‘Canada Giant’ fruits, being more intense at the later stages of shelf life, whereas the ‘Ferrovia’ fruits scored lower values even after an extended period of maintenance at 20°C (**Supplementary Figure S2A**). ‘Ferrovia’ fruits exhibited higher acceptability scores (lower index), although the pitting incidence was evident after extended exposure at room temperature (**Supplementary Figures S2B,C**).

### Respiration Rate

**FIGURE 2:**
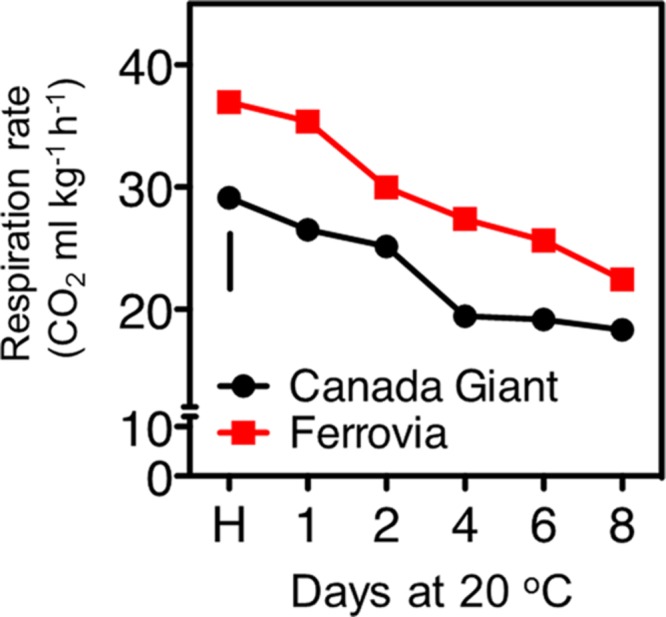
**Respiration rate of sweet cherry fruits (cvs. ‘Canada Giant’, ‘Ferrovia’) at harvest (H) and after additional maintenance at room temperature (20°C, shelf life) for 1, 2, 4, 6, and 8 days.** Each value represents the mean of three replications of 10 fruits analyzed at each time interval after harvest. The vertical bars in each particular figure represent the LSD (*P* = 0.05).

Sweet cherry is considered as a non-climacteric fruit, which is characterized by relatively high levers of respiration rate (Toivonen et al., 2004). In the current study, a gradual decrease of respiration rate was revealed for both cultivars, culminated following 8 days shelf life (**Figure 2**). ‘Ferrovia’ fruits were characterized by higher respiration rate compared to ‘Canada Giant’; given that the latter has shown lower weight losses (**Figure 1A**), no direct relationship between respiration rate and weight loss can be established by current data. It is worth to note that respiratory activity may be affected by the intensity and severity of surface pitting (**Figures 1B** and **2**); this hypothesis needs to be further elucidated.

### Textural Properties

**FIGURE 3:**
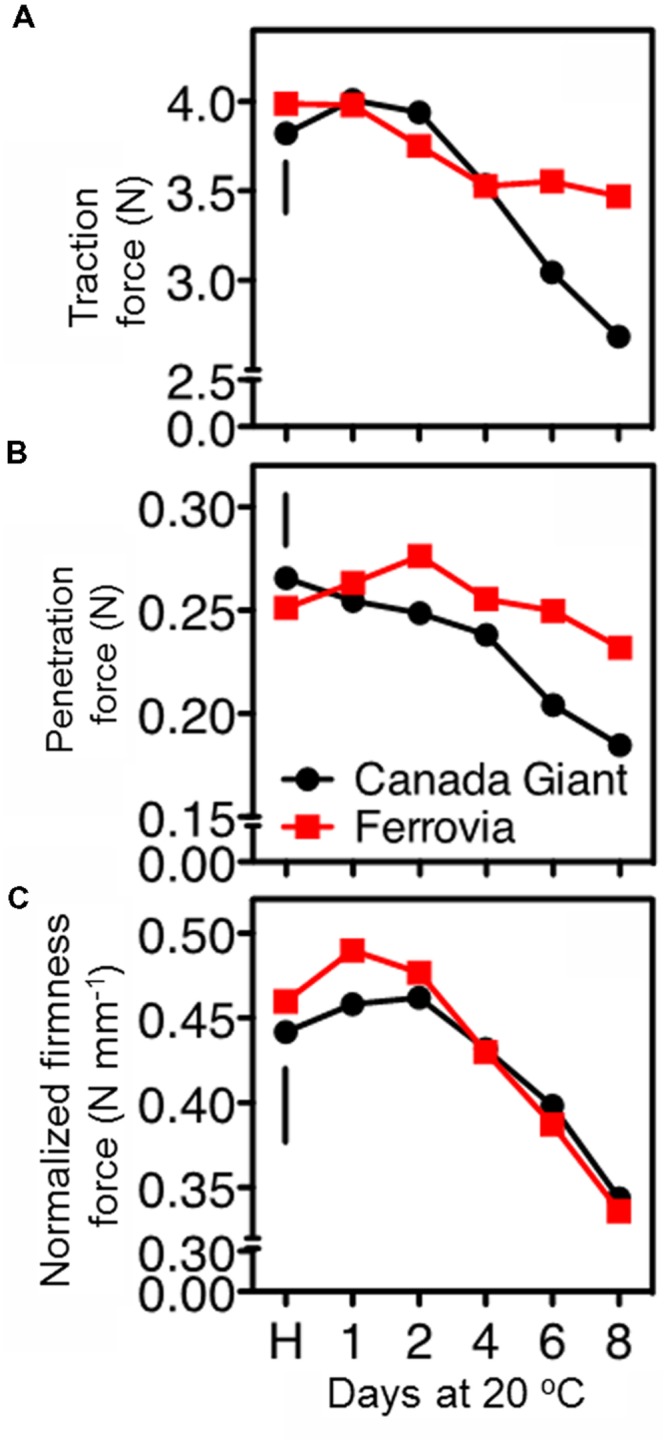
**Traction force to remove the stem of sweet cherry fruits **(A)**, skin penetration force **(B)**, and normalized firmness force at 15% fruit deformation **(C)** in sweet cherry fruits (cvs. ‘Canada Giant’, ‘Ferrovia’) at harvest (H) and after additional maintenance at room temperature (20°C, shelf life) for 1, 2, 4, 6, and 8 days.** Each value represents the mean of three replications of 10 fruits analyzed at each time interval after harvest. The vertical bars in each particular figure represent the LSD (*P* = 0.05).

The postharvest loss of quality in sweet cherries encompasses stem drying and browning (Kappel et al., 2002). Indeed, stem browning is one of the most important postharvest constraints for sweet cherry marketing (Schick and Toivonen, 2002), since water evaporates up to 14-fold more quickly from the cherry stem than from the fruit itself (Seske, 1996). In the current study, the stem removal force was similar at harvest for both cultivars; however, the long-stem ‘Ferrovia’ fruits exhibited higher titration force after prolonged shelf life period (**Figure 3A**), possibly due to less stem browning (**Supplementary Figure S2A**).

In the current study, firmness retention in the cherry fruits was evaluated using two different approaches, namely tissue penetration and tissue deformation (compression test). ‘Ferrovia’ fruits demonstrated their superiority with higher firmness values, as determined by skin penetration (**Figure 3B**), concomitant with higher acceptability (**Supplementary Figure S2C**). However, no differences between the examined cultivars were monitored when a 15% of the fruit deformation assay was applied (**Figure 3C**). These data clearly indicate the necessity to employ an array of techniques to determine textural properties of cherry fruits using both large and small deformation mechanical testing.

### Antioxidant Features of Sweet Cherry Fruits

**FIGURE 4:**
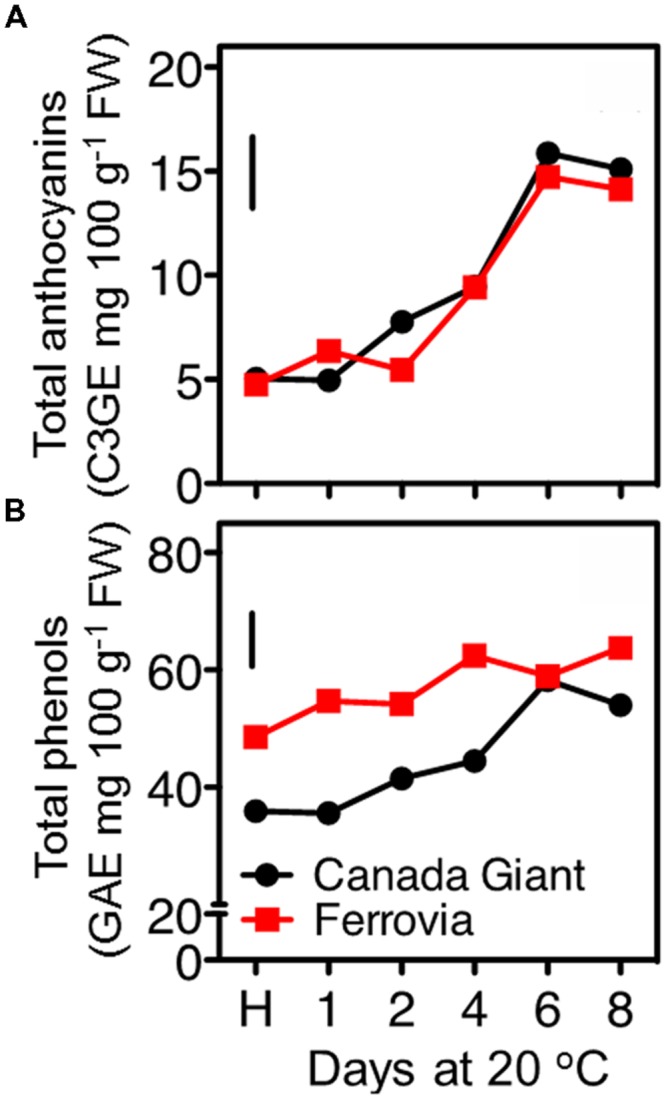
**Total anthocyanin **(A)** and total phenolic **(B)** contents of sweet cherry fruits (cvs. ‘Canada Giant’, ‘Ferrovia’) at harvest (H) and after additional maintenance at room temperature (20°C, shelf life) for 1, 2, 4, 6, and 8 days.** Each value represents the mean of three replications analyzed at each time interval after harvest. The vertical bars in each particular figure represent the LSD (*P* = 0.05).

Anthocyanin content substantially increased in both cultivars after 8 days shelf life, while generally slight differences were monitored between the examined cultivars for each storage regime (**Figure 4A**). Although sweet cherry fruit is regarded one of the richest sources of anthocyanins among fruit species (Goulas et al., 2012); however, both tested cultivars can be considered rather of low anthocyanin content compared to other studied cherry cultivars (Ballistreri et al., 2013). The major anthocyanins identified in sweet cherry fruits were the 3-*O*-glucoside and 3-*O*-rutinoside of cyanidin, with peonidin-3-*O*-rutinoside, pelargonidin-3-*O*-rutinoside being present in lower amounts (Gonçalves et al., 2007; Kelebek and Selli, 2011); differences in the predominant anthocyanins among cultivars have been recently identified (Ballistreri et al., 2013).

Among other phenolic compounds, sweet cherries are particularly rich in hydroxycinnamic acid derivatives (neochlorogenic acid, *p*-coumaroylquinic acid and chlorogenic acid; Usenik et al., 2010; Liu et al., 2011). These hydroxycinnamates are important for their potential contributions to the color of the sweet cherry fruits through the co-pigmentation process with anthocyanins (Mozetic et al., 2002), as well as they are potent antioxidants (Kopjar et al., 2012). In the current study, the total phenolics content increased upon maintenance at 20°C to certain pseudoplateau values, compared to harvest, being always higher in ‘Ferrovia’ fruit (**Figure 4B**). However, as previously mentioned for anthocyanins content, such values are regarded rather low compared to other sweet cherry cultivars (Usenik et al., 2008). An increase of total phenolic content has been also recorded during the last on-tree developmental stages (Serrano et al., 2005a); moreover, there is a further increase during maintenance at room temperature and cold storage (Diaz-Mula et al., 2012). Weight loss during postharvest storage can merely explain the accumulation of phenolic compounds.

**FIGURE 5:**
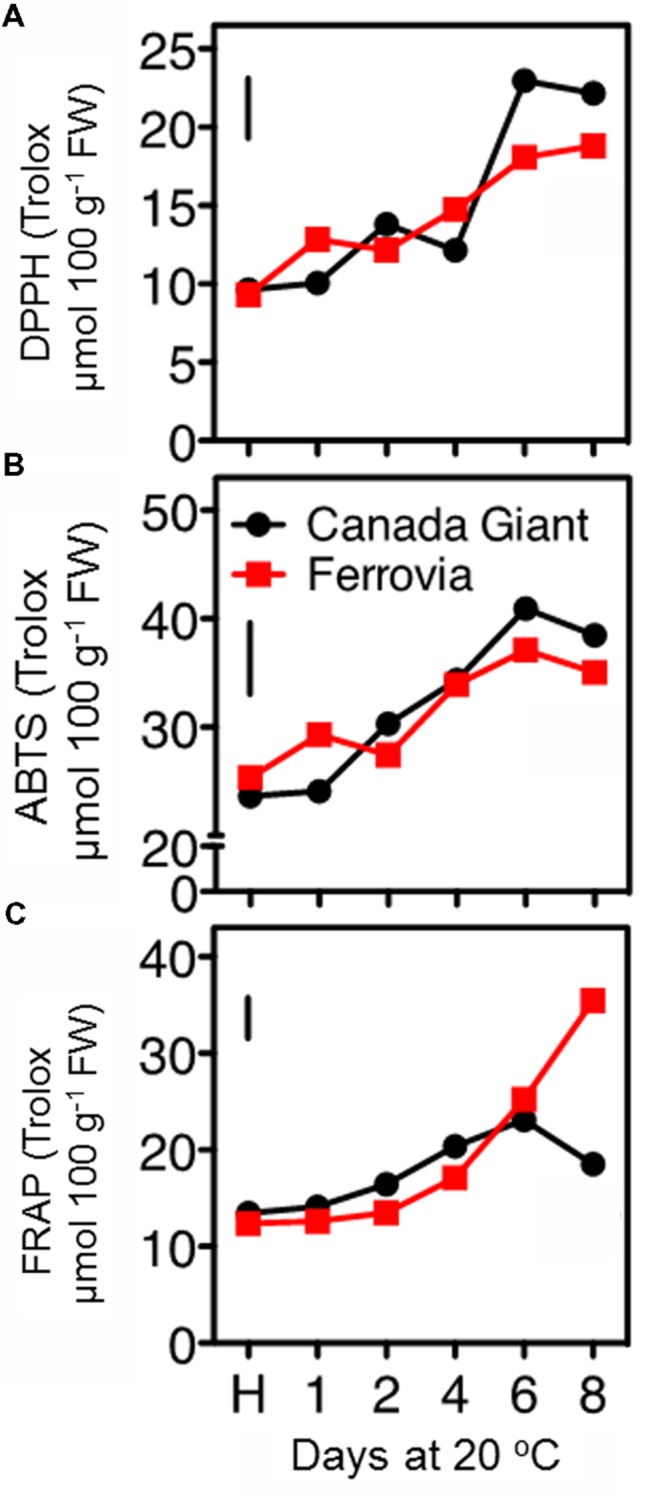
**Antioxidant capacity of sweet cherry fruits (cvs. ‘Canada Giant’, ‘Ferrovia’) at harvest (H) and after additional maintenance at room temperature (20°C, shelf life) for 1, 2, 4, 6, and 8 days, evaluated with the DPPH **(A)** ABTS radical scavenging **(B)** and FRAP assay **(C)**.** Each value represents the mean of three replications analyzed at each time interval after harvest. The vertical bars in each particular figure represent the LSD (*P* = 0.05).

In this study, the fruit antioxidant capacity was measured with three assays. All of them indicated a general induction of antioxidant capacity throughout the shelf life period (**Figures 5A–C**), concomitant with the increase monitored in total anthocyanins and phenolic contents (**Figures 4** and **5**), thus confirming previous observations (Usenik et al., 2008; Serra et al., 2011). The DPPH and ABTS assays indicated similar range of values for the examined cultivars; being slightly higher in ‘Canada Giant’ fruits after 6 and 8 days at 20°C (**Figures 5A,B**). However, this was not the case when the FRAP assay was employed; i.e., while ‘Ferrovia’ fruits demonstrated a ca. threefold increase after 8 days at 20°C, ‘Canada Giant’ fruits showed different pattern during the shelf life period and substantially lower Trolox values (**Figure 5C**). The fact that antioxidant assays used are based on different mechanisms that measure the antioxidative effect of extracts may partially explain this differentiation. Indicatively, contrasting results concerning the antioxidant potential of the ‘Ferrovia’ cultivar have been reported: Ballistreri et al. (2013) monitored rather low ORAC values for ‘Ferrovia’ among 24 examined cultivars, while Hegedus et al. (2013) ranked ‘Ferrovia’ second in antioxidant capacity among 14 cultivars, using the FRAP assay. It is interesting to note that all antioxidant assays showed a progressive increase of antioxidant capacity on both cultivars during shelf life at 20°C (**Figures 5A–C**). The latter is attributed to the accumulation of phenolic compounds and especially of anthocyanins. The antioxidant potency cannot be realized by the consumer as a quality index, but it is important to deliver fruits of high nutraceutical content since many studies reported an inverse association between risk of chronic diseases and the consumption of fruits and vegetables (Dauchet et al., 2006).

#### Metabolic Profile of Sweet Cherry by HPLC and NMR

**FIGURE 6:**
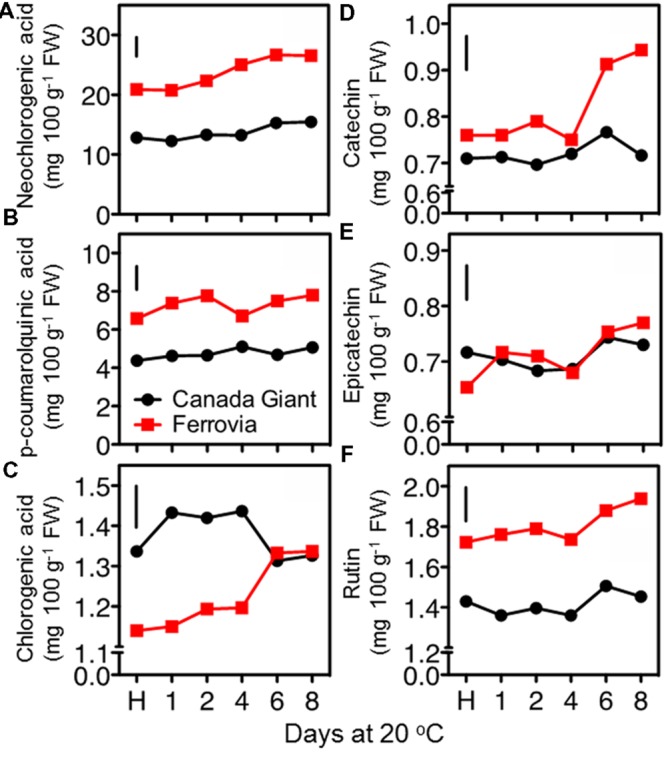
**Neochlorogenic acid **(A)**, *p*-coumaroylquinic acid **(B)**, chlorogenic acid **(C)**, catechin **(D)**, epicatechin **(E)**, and rutin **(F)** contents of sweet cherry fruits (cvs. ‘Canada Giant’, ‘Ferrovia’) at harvest (H) and after additional maintenance at room temperature (20 °C, shelf life) for 1, 2, 4, 6, and 8 days.** Each value represents the mean of three replications analyzed at each time interval after harvest. The vertical bars in each particular figure represent the LSD (*P* = 0.05).

In an attempt to rationalize the temporal changes on quality attributes and antioxidant properties of sweet cherry fruit during postharvest maintenance, we further explored their major metabolites, such as individual sugars, organic acids and phenolic compounds. The most important phenolic antioxidants in sweet cherries are anthocyanins, but the fruits also have significant amounts of colorless hydroxycinnamic acids and flavonoids (Jakobek et al., 2009). In this work, hydroxycinnamates as neochlorogenic acid and *p*-coumaroylquinic acid and flavonoids, including catechin, epicatechin and rutin, were quantified using HPLC analysis. The results showed that the sweet cherries contain higher amounts of hydroxycinnamic acid compared to flavonoids (**Figure 6**). In particular, the hydroxycinnamates/flavonoids ratios ranged between 6.5 and 10.4 in ‘Canada Giant’ and ‘Ferrovia’ fruits during postharvest ripening. An incremental change in the concentration of phenolics was found with the progress of shelf life period. A similar accumulation of hydroxycinnamates and flavonoids after 6 days storage at 15°C was also described for ‘Summit’ and ‘Saco’ sweet cherry fruit (Gonzalves et al., 2004). This phenomenon may originate from condensation of the phenolic compounds due to loss of water during ripening and/or the postharvest biosynthesis of phenolic compounds (Kalt et al., 1999). This analysis also showed that the phenolic content is highly dependent on the cultivar being considered; the sum of hydroxycinnamates and flavonoids at harvest was 21.4 mg 100 g^-1^ FW and 31.7 mg 100 g^-1^ FW for Canada Giant’ and ‘Ferrovia’ fruits, respectively. Interestingly, a significant correlation (*r* > 0.84, *p* < 0.05) was found for the estimated phenolics content by the Folin–Ciocalteu method and the HPLC assay. Thus, rapid and easy spectrophotometric method, namely Folin–Ciocalteu assay, can be initially used to give a rapid estimation of the phenolic content in sweet cherry fruit.

**FIGURE 7:**
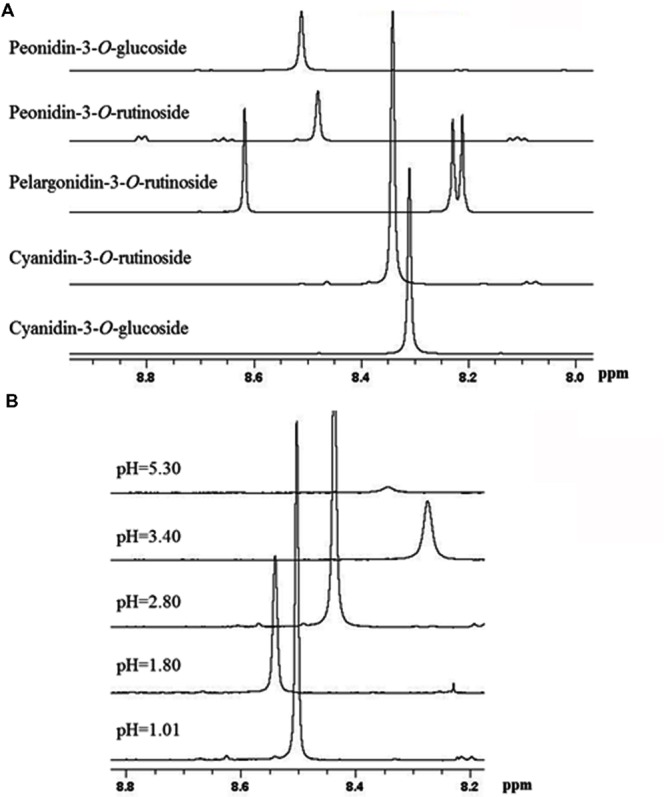
**Selected region of ^1^H-NMR spectra of anthocyanins. (A)** The effect of substitution on chemical shift of H-4 resonance of the main sweet cherry anthocyanins. **(B)** The effect of pH on the chemical shift of H-4 resonance of cyaniding-3-*O*-rutinoside.

Based on previous NMR metabolomics studies for horticultural products where methanolic extracts were used to determine primary (e.g., sugars, organic acids, and amino acids) and secondary metabolites, such as phenolic compounds (Kim et al., 2010, 2011), we have employed freeze-dried methanolic extracts in order to identify the main metabolites of sweet cherry. The freeze-dried fruit powders such as grape, raspberry, and peach are usually dissolved in D_2_O phosphate buffers at a pH ranging between 6.0 and 7.5 to obtain ^1^H-NMR spectra (Ali et al., 2011; Kim et al., 2011; Capitani et al., 2013). Taking into consideration that at pH values higher than 7.0, the anthocyanins are degraded depending on their substituent groups (Goulas et al., 2012), the optimum pH to acquire reliable ^1^H-NMR spectra for sweet cherry anthocyanins was investigated. ^1^H-NMR spectra showed that the resonance of H-4 can be used to discriminate anthocyanins in fruit extracts as it appears at 8.2-8.6 ppm, a non-overcrowded region of spectra (**Supplementary Figure S3**). **Figure 7A** also indicated that the resonance of H-4 is depended on the substitution of anthocyanin skeleton and the discrimination of anthocyanins in a complex mixture is feasible. In a next step, cyanidin-3-*O*-rutinoside was used to study the effect of pH on the chemical shift of H-4, since it is the most abundant anthocyanin in sweet cherry (Goulas et al., 2012). Data indicated that the chemical shift of the diagnostic peak (H-4) is strongly influenced by pH, highlighting the need to pH adjustment of the sample (**Figure 7B**). Finally, pH value at 3.0 was selected to obtain ^1^H-NMR spectra since a sharp peak of H-4 was recorded and additionally, it is the nearest pH to the real sweet cherry’s fruit pH at harvest.

**FIGURE 8:**
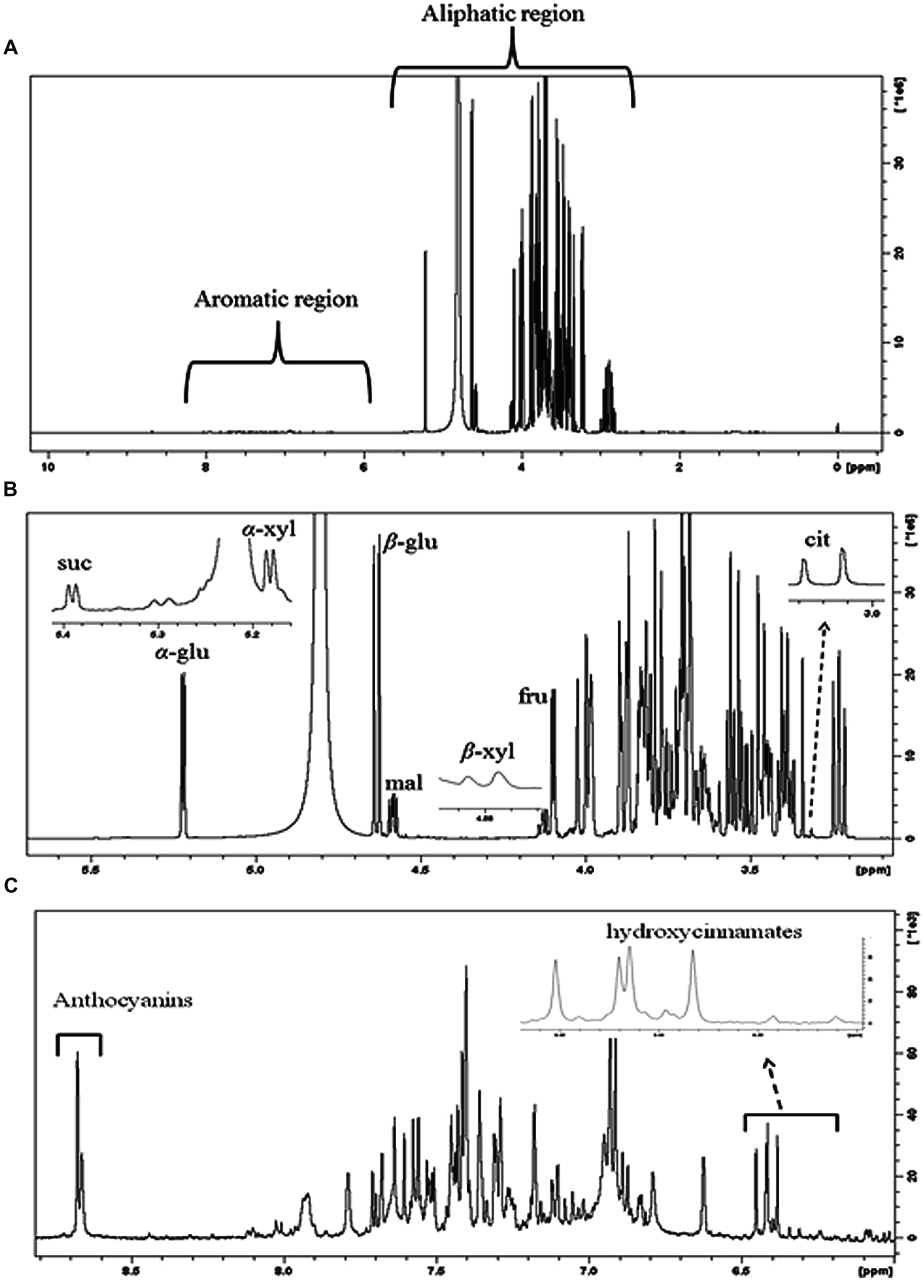
**(A)** Typical 500 MHz ^1^H-NMR spectra of “Canada Giant” sweet cherry at harvest. **(B)** Aliphatic and **(C)** aromatic region of the ^1^H-NMR spectrum. Diagnostic peaks of some metabolites are labeled. α-glu: α-glucose, β-glu: β-glucose, fru: fructose, suc: sucrose, α-xyl: α-xylose, β-xyl: β-xyl, mal: malic acid, cit: citric acid.

**Figure 8A** shows representative ^1^H-NMR spectra of sweet cherry extracts. The spectra can be divided into two regions: (i) aliphatic region where the resonances of primary metabolites, such as sugars and organic acids dominate and (ii) aromatic region where the resonances are attributed to the secondary metabolites, such as phenolic compounds. The ^1^H-NMR resonances of hydrophilic metabolites were assigned based on comparison with chemical shifts of standard compounds as well as previously published data (Gall et al., 2003; Tarachiwin et al., 2008; Kim et al., 2011; Capitani et al., 2013; Zahmanov et al., 2015). The most diagnostic resonances for the assignment of major metabolites in sweet fruits are illustrated in **Figure 8B**.

The discrimination of α- and β-glucose in the overcrowded aliphatic region of NMR spectra was performed using the resonances of the anomeric protons of glucose moieties. In particular, the resonance of anomeric proton of α- and β-glucose is displayed at 5.22 ppm (*J* = 3.80 Hz) and 4.63 ppm (*J* = 7.89 Hz), respectively. Similarly, α- and β-xylose and sucrose were identified using diagnostic doublets at 5.18, 4.57, 5.39 ppm, while the resonance of H-3 (δ = 4.11 ppm) had diagnostic importance for fructose. The NMR data indicated that β-glucose, α-glucose, and fructose were the major sugars in the examined sweet cherry cultivars, whereas the disaccharide sucrose was found at a lower concentration. The ratios of integrals α-glucose/β-glucose, *α*-glucose/fructose, and β-glucose/fructose were not affected by postharvest ripening in sweet cherry fruit. Regarding the discrimination of cultivars, we found that only the ratio of integrals α-glucose/fructose was cultivar-depended. Previous studies also reported that the abundance of sugars is strongly affected by genetic factors (Usenik et al., 2008, 2010). It is worth to notice that NMR spectroscopy allows us to discriminate the alpha and beta forms of sugars, thus providing additional information about the sweetness of sweet cherry.

Regarding the organic acids, malic acid, citric acid, shikimic, and fumaric acids were detected in sweet cherry fruits by means of diagnostic peaks. The identification of malic acid, the main organic acid, was based on a doublet of doublets resonances at 4.58 ppm (*J* = 4.54/6.88 Hz), a doublet peak at 3.04 ppm (*J* = 15.90 Hz) was used for citric acid, while the singlets at 6.85 ppm and 6.64 ppm were of diagnostic value for fumaric acid and shikimic acid.

A plethora of resonances is displayed in the aromatic region of NMR spectra. The resonances are mainly attributed to hydroxycinnamates, flavonoids and colorful anthocyanins (**Figure 8C**). Hydroxycinnamates and flavonoids were analyzed by HPLC and presented previously. owever, their identification in crude extracts using NMR could be also performed (Exarchou et al., 2002). The identification of anthocyanins by comparison of NMR spectra of extracts with these of standard compounds and literature information was impossible. A significant drift in chemical shifts of H-4 resonance in NMR spectra of extracts was observed. Co-pigmentation of anthocyanins with other organic compounds or metallic ions may be responsible for this drift in chemical shift. In this case, the detection of anthocyanins was performed by spiking with cyanidin-3-*O*-rutinoside, cyanidin-3-*O*-glucoside, peonidin-3-*O*-rutinoside, and pelargonidin-3-*O*-rutinoside into sweet cherry extracts (**Supplementary Figure S4**). Cyanidin-3-*O*-rutinoside was the major anthocyanin in both sweet cherry cultivars and its spectral line integrations were increased with the progress of postharvest ripening. Cyanidin-3-*O*-glucoside was also found, whereas peonidin-3-*O*-rutinoside and pelargonidin-3-*O*-rutinoside were not detected in any sample. The purification of sweet cherry anthocyanin extracts by removing free sugars in order to enhance spectral resolution of anthocyanin’s resonances, is recommended. Wyzgoski et al. (2010) reported the use of C_18_ SPE columns to substantially remove free sugars and to obtain NMR spectra of black raspberry anthocyanins with enhanced spectral line intensities. Overall, HPLC and NMR analyses showed mainly quantitative differences between the examined cultivars. Postharvest ripening affects both primary and secondary metabolites of sweet cherry fruit.

## Conclusion

The current study offers a comprehensive analysis of sweet cherry qualitative attributes, mechanical properties and phytochemical content. Results of the present study indicate that fruit deterioration after extended maintenance at room temperature is non-concomitant with the profile of nutraceutical properties; the latter appeared to increase with the progress of shelf life period. Further, distinct differences between the examined cultivars both at qualitative and phytochemicals content were monitored. In addition, ^1^H NMR metabolic fingerprinting was used to pinpoint and to understand the changes in quality attributes as well as in the nutraceutical content of sweet cherry. The proposed NMR protocol also allows the monitoring of primary and secondary metabolites in short experimental time, with special reference to anthocyanins. Overall, this study provides a set of common and state-of-the-art analytical tools that can be used to appraise the sweet cherry quality ambitioning to be a guide for researchers dealing with fruit sciences.

## Conflict of Interest Statement

The authors declare that the research was conducted in the absence of any commercial or financial relationships that could be construed as a potential conflict of interest.

## Supplementary Material

Table S1 **Color, expressed as L*, a* and b* values, of sweet cherry fruit (cvs. ‘Canada Giant’, ‘Ferrovia’) at harvest and additional maintenance at room temperature (20°C, shelf life) for 1, 2, 4, 6 or 8 days, respectively.** Data are the mean ± S.E (n=30).

Figure S1 **Phenotypical features of ‘Canada Giant’ **(A)** and ‘Ferrovia’ **(B)** sweet cherry fruits**.

Figure S2 **Index of stem color **(A)**, surface pitting **(B)**, and overall acceptability **(C)** of sweet cherry fruits (cvs. ‘Canada Giant’, ‘Ferrovia’) at harvest and after additional maintenance at room temperature (20°C, shelf life) for 1, 2, 4, 6, or 8 days, respectively.** Data are the mean ± SE (*n* = 30). Definition of index values is based on Feng et al. (2004).^∗^

Figure S3 **Typical structure of anthocyanidin, where the H-4 is denoted**.

Figure S4 **Selected regions of the 500 MHz 1D ^1^H NMR spectra of **(A)** cyanidin-3-*O*-rutinoside, **(B)** ‘Canada Giant’ sweet cherry, and **(C)** ‘Canada Giant’ sweet cherry with spiking of cyanidin-3-*O*-rutinoside**.
