# Supplementary material for: 1H NMR Metabolic Fingerprinting to Probe Temporal Postharvest Changes on Qualitative Attributes and Phytochemical Profile of Sweet Cherry Fruit
Source: Front Plant Sci. 2015 Nov 10;6:959. doi: 10.3389/fpls.2015.00959 (PMC4639632; doi:10.3389/fpls.2015.00959)
Supplement: Table S1 — Color, expressed as L*, a* and b* values, of sweet cherry fruit (cvs. ‘Canada Giant’, ‘Ferrovia’) at harvest and additional maintenance at room temperature (20°C, shelf life) for 1, 2, 4, 6 or 8 days, respectively. Data are the mean ± S.E (n=30). [file Table_1.DOCX]

**Supplementary Table 1**

Color, expressed as L*, a* and b* values, of sweet cherry fruit (cvs. ‘Canada Giant’, ‘Ferrovia’) at harvest and additional maintenance at room temperature (20°C, shelf life) for 1, 2, 4, 6 or 8 days, respectively. Data are the mean ± S.E (n=30)

|  | **Canada Giant** | | | | | |
| --- | --- | --- | --- | --- | --- | --- |
| Color  parameter | Shelf life period (d) | | | | | |
|  | Harvest | 1d | 2d | 4d | 6d | 8d |
| L* | 41,81±0,59a* | 40,40±0,61b | 39,29±0,47b | 37,63±0,41c | 36,00±0,33d | 35,20±0,25d |
| a* | 33,16±0,66a | 32,51±0,64a,b | 30,89±0,60b | 26,53±0,82c | 23,42±0,77d | 22,88±0,72d |
| b* | 14,74±0,61a | 12,83±0,66b | 10,94±0,50c | 8,55±0,63d | 5,66±0,45e | 5,30±0,36e |
| Chroma | 36,36±0,80a | 35,02±0,82a,b | 32,83±0,69b | 28,26±0,93c | 24,13±0,86d | 23,51±0,78d |
| Hue angle | 23,76±0,76a | 21,10±0,71b | 19,33±0,63b | 17,16±0,93c | 13,06±0,57d | 12,64±0,51d |
|  | **Ferrovia** | | | | | |
| L* | 37,03±0,37a* | 37,41±0,45a | 36,82±0,55a | 34,93±0,21b | 33,96±0,23b | 34,49±0,27b |
| a* | 28,00±0,61a,b | 30,13±0,69a | 27,15±0,82b | 23,74±0,52c | 22,75±1,22c | 22,96±0,62c |
| b* | 8,73±0,50a | 9,81±0,55a | 5,59±0,65a | 5,72±0,29b | 4,88±0,38b | 5,02±0,28b |
| Chroma | 29,38±0,72a | 31,74±0,81a,b | 28,55±0,98b | 24,44±0,57c | 23,31±1,25c | 23,52±0,66c |
| Hue angle | 16,95±0,60a | 17,69±0,61a | 16,89±0,75a | 13,30±0,41b | 11,93±0,62b | 12,69±0,37b |

*Values within rows within color parameter followed by the same letter are not significantly different

to each other at *P*=0.05 *(*Duncan’s Multiple Range test).
