# Supplementary figures and images for: 1H NMR Metabolic Fingerprinting to Probe Temporal Postharvest Changes on Qualitative Attributes and Phytochemical Profile of Sweet Cherry Fruit
Source: Front Plant Sci. 2015 Nov 10;6:959. doi: 10.3389/fpls.2015.00959 (PMC4639632; doi:10.3389/fpls.2015.00959)

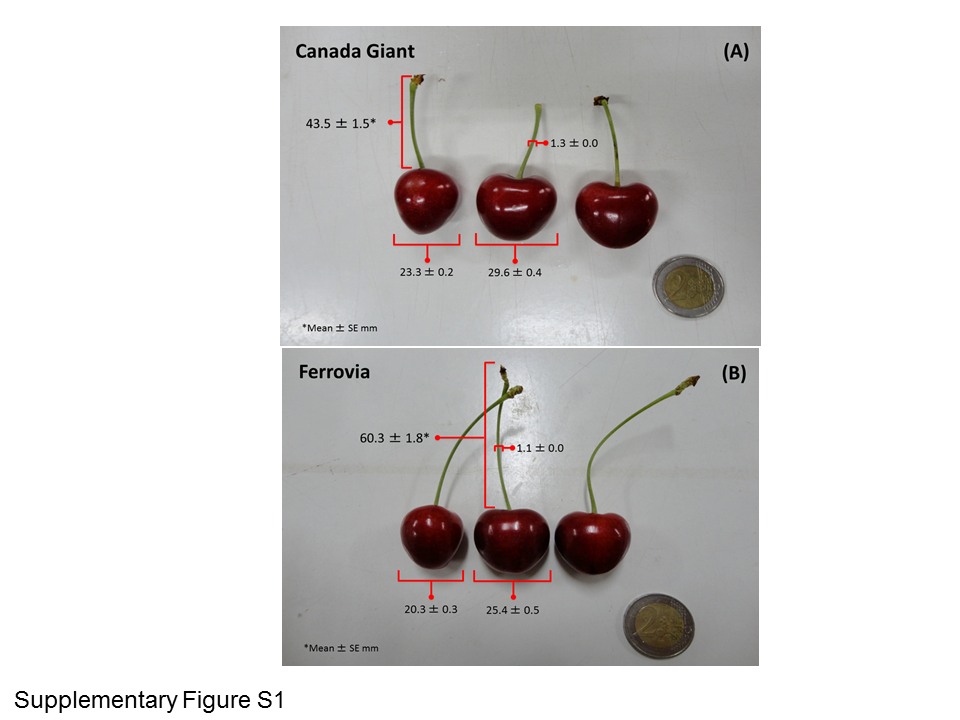

Supplement: Figure S1 — Phenotypical features of ‘Canada Giant’ (A) and ‘Ferrovia’ (B) sweet cherry fruits. [file Image_1.TIF]

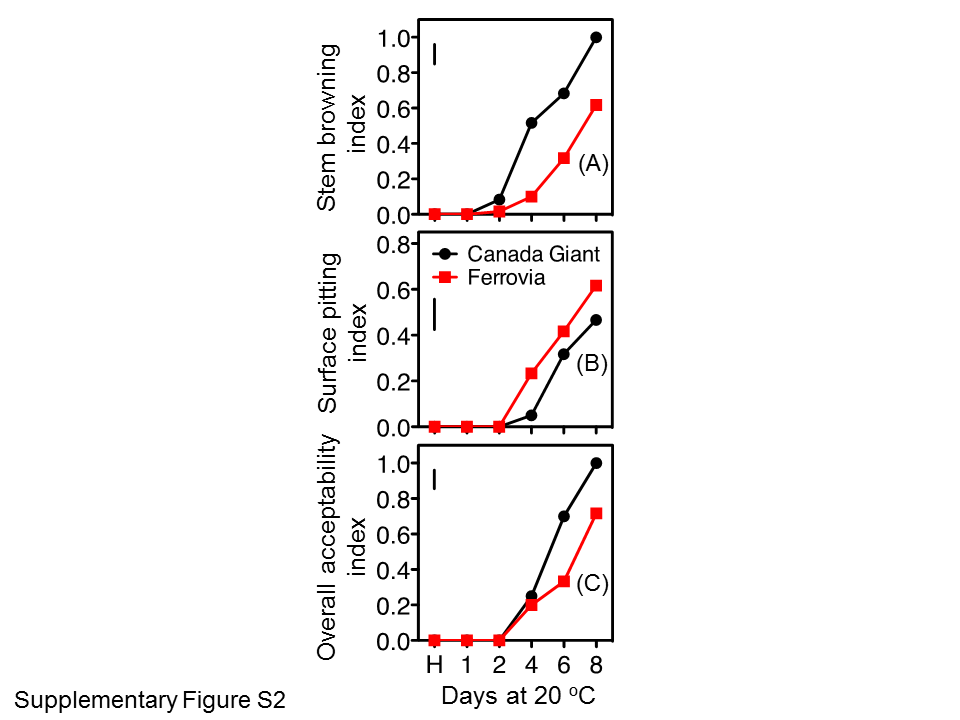

Supplement: Figure S2 — Index of stem color (A), surface pitting (B), and overall acceptability (C) of sweet cherry fruits (cvs. ‘Canada Giant’, ‘Ferrovia’) at harvest and after additional maintenance at room temperature (20°C, shelf life) for 1, 2, 4, 6, or 8 days, respectively. Data are the mean ± SE (n = 30). Definition of index values is based on Feng et al. (2004).∗ [file Image_2.TIF]

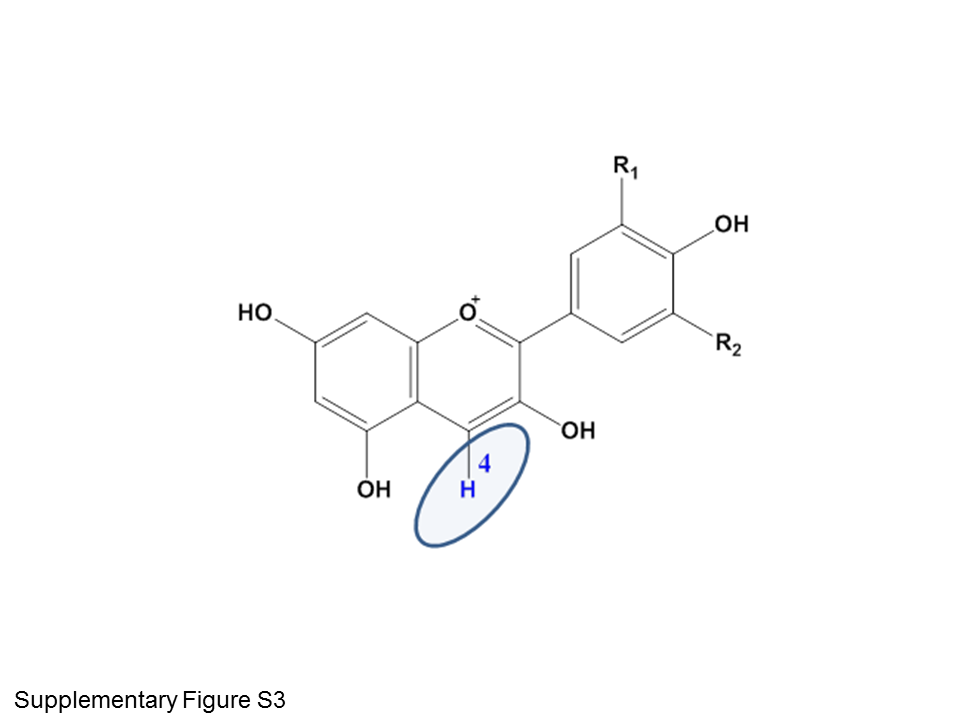

Supplement: Figure S3 — Typical structure of anthocyanidin, where the H-4 is denoted. [file Image_3.TIF]

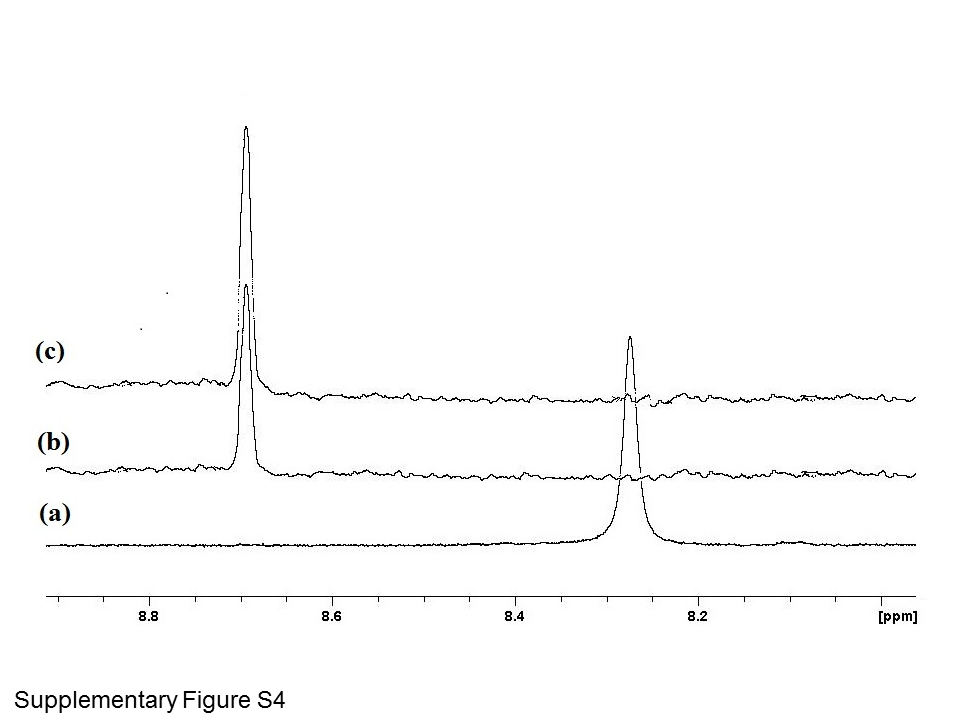

Supplement: Figure S4 — Selected regions of the 500 MHz 1D 1H NMR spectra of (A) cyanidin-3-O-rutinoside, (B) ‘Canada Giant’ sweet cherry, and (C) ‘Canada Giant’ sweet cherry with spiking of cyanidin-3-O-rutinoside. [file Image_4.TIF]
